# Real-time automatic prediction of treatment response to transcatheter arterial chemoembolization in patients with hepatocellular carcinoma using deep learning based on digital subtraction angiography videos

**DOI:** 10.1186/s40644-022-00457-3

**Published:** 2022-05-12

**Authors:** Lu Zhang, Yicheng Jiang, Zhe Jin, Wenting Jiang, Bin Zhang, Changmiao Wang, Lingeng Wu, Luyan Chen, Qiuying Chen, Shuyi Liu, Jingjing You, Xiaokai Mo, Jing Liu, Zhiyuan Xiong, Tao Huang, Liyang Yang, Xiang Wan, Ge Wen, Xiao Guang Han, Weijun Fan, Shuixing Zhang

**Affiliations:** 1 Department of Radiology, The First Affiliated Hospital of Jinan University, No. 613 Huangpu West Road, Tianhe District, Guangzhou, 510627 Guangdong China; 2 Shenzhen Research Institute of Big Data, Shenzhen, Guangdong China; 3 School of Science and Engineering, The Chinese University of Hong Kong, 2001 Longxiang Avenue, Longgang District, Shenzhen, Guangdong China; 4 Department of Interventional Therapy, The Second Affiliated Hospital of Guangzhou University of Traditional Chinese Medicine, Guangzhou, Guangdong China; 5 Department of Minimally Invasive Intervention, Sun Yat-sen University Cancer Center; State Key Laboratory of Oncology in South China; Collaborative Innovation Center for Cancer Medicine, Guangzhou, Guangdong China; 6 Medical Imaging Center, Nanfang Hospital, Southern Medical University, 1023 Shatai South Road, Baiyun District, Guangzhou, Guangdong China

**Keywords:** Hepatocellular carcinoma, Transcatheter arterial chemoembolization, Deep learning, DSA videos

## Abstract

**Background:**

Transcatheter arterial chemoembolization (TACE) is the mainstay of therapy for intermediate-stage hepatocellular carcinoma (HCC); yet its efficacy varies between patients with the same tumor stage. Accurate prediction of TACE response remains a major concern to avoid overtreatment. Thus, we aimed to develop and validate an artificial intelligence system for real-time automatic prediction of TACE response in HCC patients based on digital subtraction angiography (DSA) videos via a deep learning approach.

**Methods:**

This retrospective cohort study included a total of 605 patients with intermediate-stage HCC who received TACE as their initial therapy. A fully automated framework (i.e., DSA-Net) contained a U-net model for automatic tumor segmentation (Model 1) and a ResNet model for the prediction of treatment response to the first TACE (Model 2). The two models were trained in 360 patients, internally validated in 124 patients, and externally validated in 121 patients. Dice coefficient and receiver operating characteristic curves were used to evaluate the performance of Models 1 and 2, respectively.

**Results:**

Model 1 yielded a Dice coefficient of 0.75 (95% confidence interval [CI]: 0.73–0.78) and 0.73 (95% CI: 0.71–0.75) for the internal validation and external validation cohorts, respectively. Integrating the DSA videos, segmentation results, and clinical variables (mainly demographics and liver function parameters), Model 2 predicted treatment response to first TACE with an accuracy of 78.2% (95%CI: 74.2–82.3), sensitivity of 77.6% (95%CI: 70.7–84.0), and specificity of 78.7% (95%CI: 72.9–84.1) for the internal validation cohort, and accuracy of 75.1% (95% CI: 73.1–81.7), sensitivity of 50.5% (95%CI: 40.0–61.5), and specificity of 83.5% (95%CI: 79.2–87.7) for the external validation cohort. Kaplan-Meier curves showed a significant difference in progression-free survival between the responders and non-responders divided by Model 2 (*p* = 0.002).

**Conclusions:**

Our multi-task deep learning framework provided a real-time effective approach for decoding DSA videos and can offer clinical-decision support for TACE treatment in intermediate-stage HCC patients in real-world settings.

**Supplementary Information:**

The online version contains supplementary material available at 10.1186/s40644-022-00457-3.

## Background

Hepatocellular carcinoma (HCC) is the sixth most common malignant cancer and the fourth leading cause of cancer-related deaths worldwide [1]. Less than 30% of HCC patients receive potentially curative therapies (e.g., resection, ablation therapy, and liver transplantation), and most patients diagnosed with intermediate- and advanced-stage HCC only receive unresectable therapy [2]. According to international guidelines, transcatheter arterial chemoembolization (TACE) is currently the standard treatment to manage patients with intermediate- and unresectable early-stage HCC and improve patient survival rates [3, 4]. However, intermediate-stage HCC is determined based on liver function and tumor burden, which results in heterogeneous outcomes, such as varied treatment response of 15–55% and median overall survival of 13–43 months [1, 2]. In addition, some patients may experience deterioration of liver function after TACE, which may negatively impact prognosis and potentially impede consequent anti-tumor treatments if patients’ liver function further exacerbates due to repeated TACE cycles [3, 4]. Thus, a pre-procedure prediction model to estimate treatment response to TACE as a reference may aid in clinical decision-making and thus, enable patients to achieve acceptable therapeutic efficacy.

Digital subtraction angiography (DSA) is an indispensable procedure during TACE therapy that dynamically provides information on lesion location, catheter navigation, arterial blood supply, and treatment assessment in real-time, which influence the diagnosis and treatment of most HCC patients who receive TACE [5]. Given the impact of DSA on TACE diagnosis and treatment, technological advances have been made to improve image quality, and computer-aided software and 3D-angiography have been introduced to improve interprocedural guidelines. However, despite these advances, the location and evaluation of lesions in DSA videos are still reliant on operators’ subjectivity, and heterogeneity exists in terms of techniques and treatment assessment, which can lead to different outcomes in HCC patients. Thus, there is a crucial need for quantitative analysis of DSA videos, especially for the objective evaluation of treatment response.

Key advances in mining medical images for information have been made in recent years. Machine learning, especially deep learning (DL), has been used to extract more information from images than what can be observed by radiologists. DL-based models have been widely applied in HCC, such as for tumor segmentation [6], differential diagnosis [7], and prognosis [8]. However, reports of using DL-based models for predicting treatment response in HCC patients treated with TACE are scarce, and to the best of our knowledge, only two studies have been conducted recently [9, 10]. Notably, the models in these studies were constructed based on pretherapy contrast-enhanced ultrasound (US) or computed tomography (CT) images, whereas the efficacy of TACE depends primarily on arterial blood supply and tumor burden, which can be directly observed by angiography during TACE [11, 12]. Currently, DL-based models using DSA images are only used to detect and segment vascular diseases, such as coronary artery stenosis and intracranial aneurysm [13, 14].

Thus, we aimed to propose a DL architecture, called DSA-Net, which incorporates clinical variables and decoded DSA information to aid clinicians in making personalized treatment decisions and identifying ideal candidates for TACE. The DSA-Net consists of a DL-based model for the tumor segmentation on DSA videos and a DL-based model for treatment response prediction.

## Materials and methods

### Patients and datasets

**Fig. 1:**
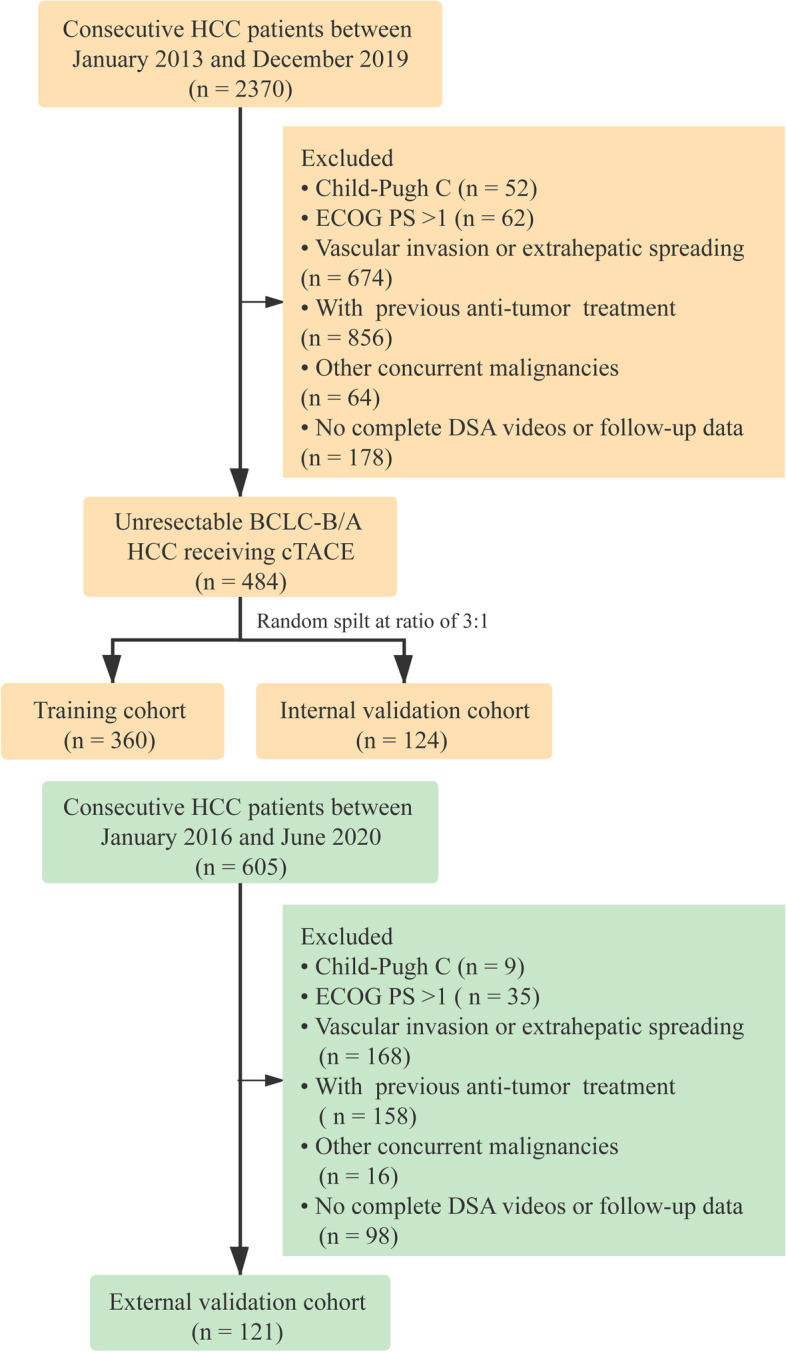
Flowchart of patient inclusion/exclusion for two centers

This retrospective study was approved by the institutional research ethics committee of participating hospitals, and the need for informed consent was waived. Consecutive patients with newly diagnosed HCC treated with conventional TACE (cTACE) were retrospectively reviewed. Diagnosis of HCC was based on either histology or dynamic imaging (CT/magnetic resonance imaging [MRI]) evaluations according to the American Association for the Study of Liver Diseases (AASLD) or European Association for the Study of the Liver (EASL) guidelines [15, 16]. Inclusion criteria were: (1) aged 18 years or older; (2) unresectable Barcelona Clinic Liver Cancer (BCLC) stage A/B; (3) no previous anti-tumor treatment. Exclusion criteria were: (1) Child-Pugh C liver function or evidence of hepatic decompensation, including refractory ascites, esophageal or gastric variceal bleeding, or hepatic encephalopathy; (2) Eastern Cooperative Oncology Group (ECOG) performance score of > 1; (3) no complete DSA videos or follow-up data; (4) diagnosis or history of any other concurrent malignancies. Baseline CT/MRI was performed 5–7 days before the first TACE session and response assessment imaging was performed approximately 4–6 weeks after TACE (before the subsequent therapy session). The flowchart of patient inclusion is shown in Fig. 1. The primary cohort contained 484 consecutive HCC patients who were newly diagnosed between January 14, 2013 and December 24, 2019. The primary dataset was randomly divided into training (*n* = 360) and internal validation cohorts (*n* = 124) at a ratio of 3:1. The external validation cohort was composed of 121 HCC patients who were diagnosed between January 29, 2016 and June 10, 2020.

Clinical characteristics included age, sex, hepatitis B virus (HBV), a-Fetoprotein (AFP), prothrombin time (PT), and liver function parameters, which included Child–Pugh score, ascites, total bilirubin (TBIL), albumin (ALB), aspartate aminotransferase (AST), alanine aminotransferase (ALT), and C-reactive protein (CRP). All laboratory data were obtained within the 3 days before the first TACE session.

### TACE procedure

Before the TACE procedure, we performed routine DSA of the superior mesenteric and hepatic arteries. During the TACE procedure, the interventional radiologists super-selectively administrated chemotherapeutic drugs (10–50 mg doxorubicin or epirubicin) mixed with lipiodol (5–20 ml) through feeding arteries until arterial flow stasis was observed. Subsequently, we embolized the feeding arteries using a gelatin sponge or polyvinyl alcohol foam particles, as observed on angiography. Each procedure was performed by interventional radiologists with more than 8 years of experience. If patients had a favorable clinical status and laboratory findings and there was no evidence of extrahepatic spread or major portal vein invasion, sequential TACE was performed on an “on-demand” basis in cases where residual viable tumors were found in follow-up CT/MRI every 4–8 weeks after each TACE session.

### Study endpoints

The primary endpoint of this study was treatment response after the first TACE session (approximately 4–6 weeks after TACE), which was assessed according to the modified Response Evaluation Criteria in Solid Tumors (mRECIST) [17] by two radiologists with more than 5 years of experience in liver imaging and checked by one interventional radiologist with 8 years of experience in TACE therapy. When there was any ambiguity in tumor response assessment, the final classification was made by observers’ consensus. Patients were divided into two groups: 1) responders, who were patients who initially achieved an objective response to the first TACE session (defined as those assessed as having complete response [CR] or partial response [PR]), and 2) non-responders, who were patients who did not achieve an objective response during the treatment course (those assessed as having stable disease [SD] or progressive disease [PD]). The secondary endpoint was 3-year progression-free survival (PFS), which was defined as the time from the initial TACE to disease progression or death from any cause.

### Imaging acquisition and annotation

We obtained pre-TACE angiography of the proper or branch hepatic artery from portable network graphics (PNG) images or audio-video interleaved (AVI) videos formats from the Picture Archiving and Communication Systems (PACS). Each DSA video contained 20–30 frames with 1021 × 788 pixel-wise resolution. For further segmentation, all DSA AVI videos were first transformed into consecutive PNG images. We also acquired pre- and post-therapy CT/MRI digital imaging and communications in medicine images from PACS to assist in determining the tumor location, segmenting the tumor on DSA videos, and assessing treatment response. DSA videos acquisition parameters are described in Supplementary Method S1*.*

**Fig. 2:**
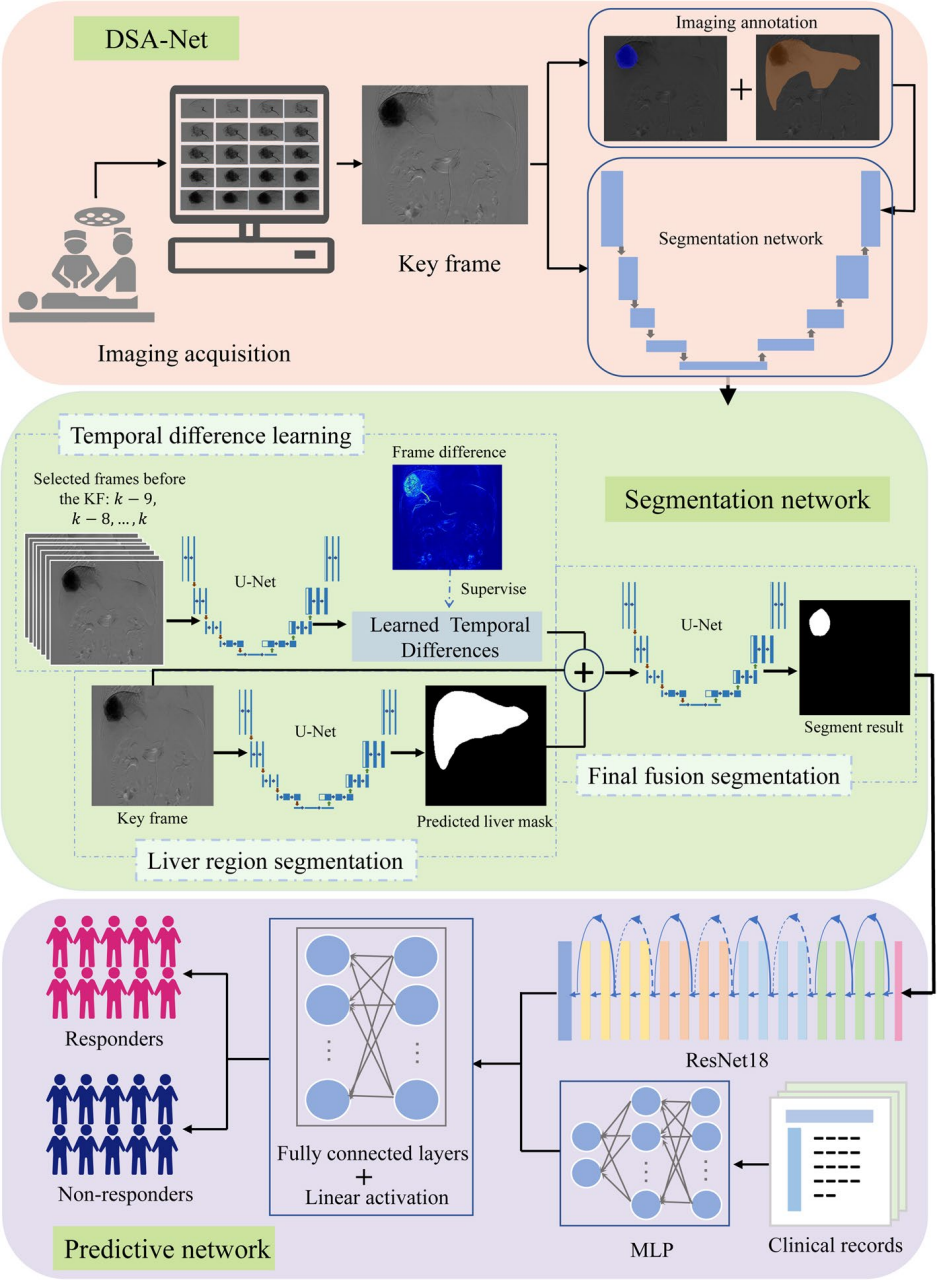
Workflow of DSA-Net. The procedure of DSA-Net contains imaging acquisition, key frame selection, and construction of segmentation network and prediction network. The segmentation network consists of a temporal difference learning module, a liver region segmentation sub-network, and a final fusion segmentation sub-network. The prediction network included a ResNet18 for image data and a multi-layer perceptron for tabular data

The tumor and whole liver were manually delineated on DSA images by two experienced radiologists using the Labelme software (http://labelme.csail.mit.edu), which was checked by one experienced interventional radiologist. We selected the frame in the DSA videos with full staining of the tumor or arterial flow stasis as the key frame. For data augmentation, we selected two further consecutive frames (i.e., the key frame and the frames before and after the key frame) for training the segmentation model. We also conducted the experiment with the original key frame selected from each DSA video for training, which was slightly poorer than using our current data augmentation. The flowchart of model construction is shown in Fig. 2.

### DSA-net construction

Before model construction, we preprocessed the DSA images. Firstly, in the clinic, interventional radiologists select DSA video frames with full staining of tumors or arterial flow stasis for the diagnosis and measurement of tumor parameters. Thus, we defined such frames as key frames to simulate the human diagnosis process. Specifically, we designed a simple method for automatically selecting key frames (Supplementary Method S2). Second, because the black borders around the raw DSA images usually negatively impact tumor segmentation, we used median filtering to remove the noise in the black border and thresholding to detect and remove the black borders. Third, to unify the gray value range of the images, a traditional min-max normalization was applied to the DSA images. Lastly, we used torchvision.transforms. Resize to unify the resolution of images to 256 × 256.

We constructed a DSA-Net including a segmentation network (Model 1) for the automatic tumor segmentation on DSA videos, and a treatment response prediction network (Model 2) for evaluating treatment response to the first TACE session.

### Segmentation network (model 1)

Initially, we trained our baseline model on the key frames using U-Net [18], U-Net++ [19], nnU-Net [20], Attention U-Net [21], and U^2^-Net [22]. Given the convenience of the implementation and modification of the model, we chose the U-Net as the backbone of our final model.

Considering the specificity of DSA videos, we proposed the novel Model 1 for HCC segmentation, which included a temporal difference learning (TDL) module, a liver region segmentation (LRS) sub-network, and a final fusion segmentation (FFS) sub-network (Supplementary Method S2). The three inputs of FFS were the key frames, the liver region masks predicted by LRS, and the temporal difference learned by TDL. The key frames (256 × 256 × 1), alongside the learned temporal difference (256 × 256 × 1) by TDL and the predicted liver region masks (256 × 256 × 1) by LRS, were concatenated and fed into this network. Finally, we co-trained the TDL and LRS networks simultaneously with the segmentation of U-Net. The segmentation model was developed on the training cohort using five-fold cross-validation, which was optimized on the validation cohort and evaluated on the testing cohort. The loss functions were defined as follows:

1 $${L}_{LTD}={\left|{I}_{LTD}-{I}_{FD}\right|}_{\mathrm{L}1}$$

2 $${L}_{LRS}=\mathrm{a}\ast {\left|{I}_{LRS}-{I}_{LM}\right|}_{BCE}+\left(1-\mathrm{a}\right)\ast {\left|{I}_{LRS}-{I}_{LM}\right|}_{DICE\kern0.5em }$$

3 $${L}_{seg}=\mathrm{a}\ast {\left|{I}_{seg}-{I}_{GT}\right|}_{BCE}+\left(1-\mathrm{a}\right)\ast {\left|{I}_{seg}-{I}_{GT}\right|}_{DICE}$$

4 $${L}_{Total=}{\lambda}_0\ast {L}_{LTD+}{\lambda}_1\ast {L}_{LRS}+{L}_{seg}$$

where L_LTD_, L_LRS_, and L_seg_ denote the losses of learned time difference (LTD), LRS, and tumor segmentation, respectively, and L_Total_ is the total loss of the whole network. Note that, a, λ0, λ1 are hyperparameters that were used to control the effect of the loss function and were experimentally set to 0.5, 0.1, and 1, respectively. FD is the frame differences, LM is the liver region masks, and GT is the ground truth.

We analyzed the potential factors that would affect the automatic segmentation, including lesion size, lesion number, and the surrounding inference images. Because some operators habituate to enlarge DSA images, we also compared the performance between different fields of view (FOVs).

### Treatment response prediction network (model 2)

The tumor areas acquired from Model 1 and clinical variables were applied to construct Model 2 for predicting treatment response to the first TACE session. For the classification task, we constructed a model containing two branches: one convolutional neural network (CNN) subnet based on ResNet18 for our image data and a multi-layer perceptron for our tabular data [23]. The preprocessed procedures of tabular data are described in Supplementary Method S2*.* The outputs of the two branches, which referred to the features extracted from the image and tabular data, respectively, were combined and fed into the final linear layers to obtain the final binary class to predict treatment response (Supplementary Method S2). Consequently, a series of comparative experiments were conducted by changing the input and using the original key frames and ground truth to train the predictive model as the upper bound of the whole model.

### Statistical analysis

The clinical characteristics between cohorts were compared using independent t-tests (or Mann–Whitney U test as appropriate) for continuous variables and χ2 tests (or Fisher exact test, as appropriate) for categorical variables. The interobserver agreement of treatment response evaluation was measured by the intraclass correlation coefficient (ICC); an ICC > 0.75 was regarded as good [24]. We used the Dice coefficient, accuracy, patient-level sensitivity, specificity, positive predictive value (PPV), negative predictive value (NPV), lesion-level sensitivity, and false-positive ratio (FPR) to assess the performance of Model 1. The 95% confidence intervals (CIs) were obtained using bootstrapping to assess variability. The performance of Model 2 was evaluated by an area under the receiver operating characteristic curve, along with accuracy, sensitivity, specificity, PPV, and NPV. The performance of models was compared using the Delong’s test. The PFS between the responders and non-responders was compared using the Kaplan-Meier curve and the log-rank test. All statistical tests were two-sided, and *p* < 0.05 indicated statistical significance. Statistical analyses were performed using SPSS software (version 22.0, SPSS Statistics, Armonk, NY, USA) and Python (version 3.8.3).

## Results

### Baseline clinical characteristics

A total of 605 eligible patients with 610 DSA videos (three patients with two DSA videos and one patient with three DSA videos) and 905 lesions from two hospitals were included for analysis. Baseline clinical characteristics of the training, internal validation, and external validation cohorts are presented in Table 1. There were no significant differences in any of features between the training cohort and internal validation cohort. The ICC of treatment response evaluation between two observers ranged from 0.930 to 0.944.

**Table 1:** Baseline characteristics of the training and validation cohorts

Variables	All patients (*n* = 605)	Training cohort (*n* = 360)	Internal validation cohort (*n* = 124)	External validation cohort (*n* = 121)
Age (years)	55.0 ± 11.9	55.06 ± 12.2	55.5 ± 11.6	54.5 ± 11.3
Sex (male)	548 (90.6)	330 (91.7)	107 (86.3)	111 (91.7)
HBV +	530 (87.6)	306 (85.0)	107 (86.3)	112 (92.6)
BCLC stage				
A	67 (11.1)	36 (10.0)	16 (12.9)	15 (12.4)
B	538 (88.9)	324 (90.0)	108 (87.1)	106 (87.6)
Child-Pugh score				
5	437 (72.2)	263 (73.1)	100 (80.6)	74 (61.2)
6	106 (17.5)	62 (17.2)	15 (12.1)	29 (24.0)
7	38 (6.3)	21 (5.8)	8 (6.5)	9 (7.4)
8	17 (2.8)	10 (2.8)	2 (1.6)	7 (5.8)
9	7 (1.2)	4 (1.1)	1 (0.8)	2 (1.7)
Ascites	43 (7.1)	24 (6.7)	7 (5.6)	12 (9.9)
PT (s)	12.8 ± 5.9	13.1 ± 7.6	12.3 ± 1.3	12.5 ± 1.6
TBIL (μmol/L)	18.6 ± 16.6	19.1 ± 18.6	16.7 ± 8.7	17.5 ± 13.4
ALB (g/L)	40.7 ± 23.9	41.8 ± 30.7	40.2 ± 4.7	38.0 ± 5.3
AST (≥40, IU/L)	351 (58.0)	230 (63.9)	69 (55.6)	52 (43.0)
ALT (≥40, IU/L)	311 (51.4)	198 (55.0)	69 (55.6)	44 (36.4)
CRP (≥1, mg/L)	313 (86.9)	313 (86.9)	108 (87.1)	96 (79.3)
AFP (≥200, ng/ml)	267 (44.1)	172 (47.8)	51 (41.1)	44 (36.4)
Treatment response				
Responders	335 (55.4)	176 (48.9)	69 (55.6)	90 (74.4)
Non-responders	270 (44.6)	184 (51.1)	55 (44.4)	31 (25.6)
Combined with other treatment (yes)	388 (64.1)	237 (65.8)	81 (65.3)	70 (57.9)
Rounds of TACE (≥2)	523 (86.4)	320 (88.9)	104 (83.9)	99 (81.8)

Qualitative variables are in n (%) and quantitative variables are in mean ± SD, when appropriate. *HBV* Hepatitis B virus, *BCLC* Barcelona Clinic Liver Cancer, *AFP* a-Fetoprotein, *PT* Prothrombin time, *TBIL* Total bilirubin, *ALB* Albumin, *AST* Aspartate aminotransferase, *ALT* Alanine aminotransferase, *CRP* C-reactive protein

### Performance of segmentation network (model 1)

Among the baseline segmentation models, the nnU-net had a slightly higher Dice coefficient of 0.72 (95% CI: 0.70–0.74) (Supplementary Table S1). U-net was selected as the baseline model for further modification, which had a Dice coefficient of 0.71 (95% CI: 0.68–0.74). Then, according to the learned temporal difference of the frames, the performance of the segmentation model based on the baseline model plus TDL was effectively boosted. The Dice coefficient increased from 0.71 to 0.72. The performance of TDL with different frames was slightly lower than that the 10 frames, and thus the 10 frames of TDL were used in subsequent analyses (Supplementary Table S2). Similarly, the LRS was added into the baseline model and had higher performance compared with that of the baseline model and TDL. The Dice coefficient increased from 0.71 to 0.73.

**Fig. 3:**
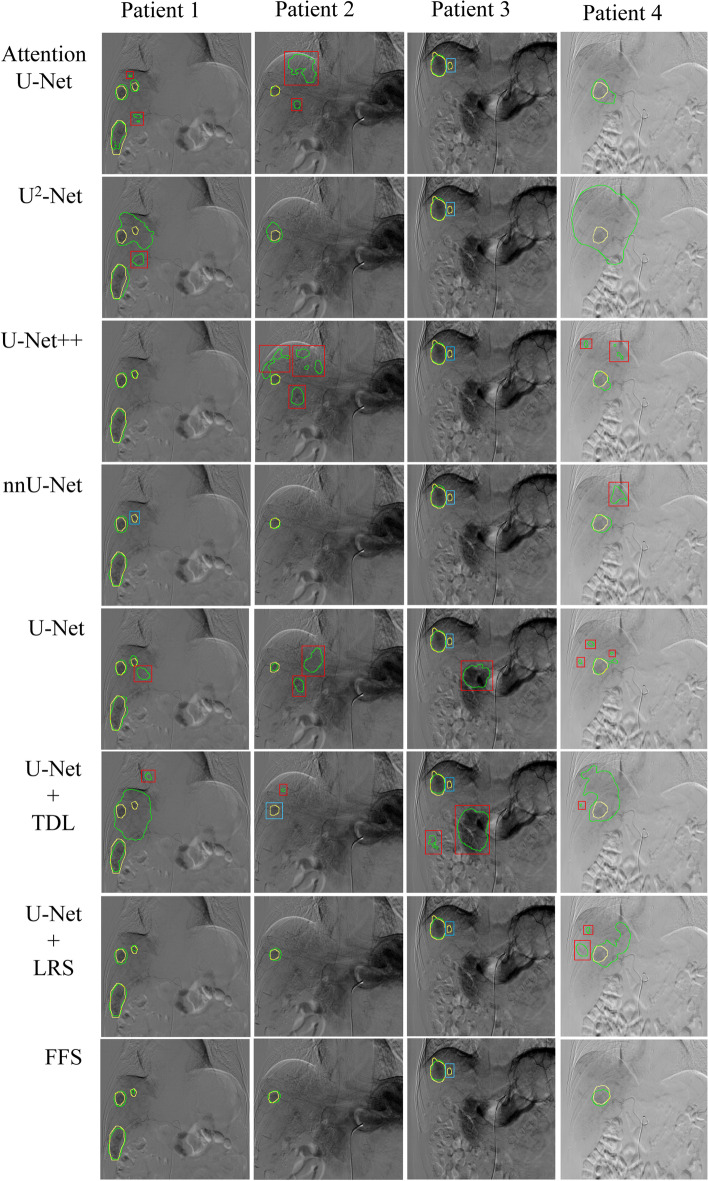
A comparison of image segmentation algorithms in the validation cohorts. Ground truth and predicted mask of tumors are labeled in yellow and cyan-blue, respectively. Compared with other algorithms, the FFS model achieved the lowest false positive and missed segmentation in the following four situations: multiple lesions (patient 1), a small lesion < 3 cm (patient 2), a small lesion < 3 cm with obvious surrounding stomach and intestine images (patient 3), and poor image quality (patient 4). TDL, temporal difference learning; LRS, liver region segmentation; FFS, final fusion segmentation

Finally, a final fusion segmentation Model 1 was built by integrating the baseline model with TDL and LRS. In the internal validation cohort, Model 1 achieved a Dice coefficient, accuracy, patient-level sensitivity, specificity, PPV, NPV, lesion-level sensitivity, and FPR of 0.75 (95% CI: 0.73–0.78), 97.1% (95% CI: 96.8–97.5), 82.3% (95% CI: 79.8–84.8), 98.4% (95% CI: 98.1–98.6), 77.9% (95% CI: 75.0–80.6), 98.3% (95% CI: 97.9–98.6), 87.2% (95% CI: 84.4–89.9), and 23.8% (95% CI: 20.4–27.3), respectively. Furthermore, an independent external validation cohort was used to test the generalizability and robustness of Model 1, which contained 121 patients with 122 DSA videos. The model achieved a Dice coefficient, accuracy, patient-level sensitivity, specificity, PPV, NPV, lesion-level sensitivity, and FPR of 0.73 (95% CI: 0.71–0.75), 97.1% (95% CI: 96.7–97.4), 79.0% (95% CI: 76.4–81.1), 98.7% (95% CI: 98.5–98.9), 79.6% (95% CI: 76.9–82.0), 98.1% (95% CI: 97.8–98.4), 94.3% (95% CI: 92.4–96.2), and 30.8% (95% CI: 27.9–33.7), respectively. The model dealt with videos in 10 ms. The performance of the segmentation models is shown in Table 2 and Fig. 3.

**Table 2:** Performance of segmentation models in the validation cohorts

Cohort	Model	Dice	Accuracy	Patient-level sensitivity	Specificity	PPV	NPV	Lesion-level sensitivity	FPR
Internal validation cohort	Baseline	0.71 (0.68–0.74)	96.8 (96.4–97.2)	80.2 (77.6–82.7)	98.2 (97.8–98.5)	75.5 (72.3–78.5)	98.1 (97.8–98.4)	84.8 (81.9–87.8)	34.0 (30.5–37.4)
	Baseline + TDL	0.72 (0.70–0.75)	96.7 (96.3–97.0)	83.3 (81.0–85.6)	97.7 (97.3–98.0)	73.4 (70.2–76.3)	98.4 (98.2–98.7)	81.8 (78.6–85.0)	32.2 (28.8–35.5)
	Baseline + LRS	0.73 (0.70–0.76)	97.0 (96.6–97.4)	80.0 (77.3–82.8)	98.5 (98.2–98.7)	75.5 (72.3–78.4)	98.0 (97.7–98.4)	83.2 (80.1–86.3)	22.7 (19.3–26.1)
	FFS	0.75 (0.73–0.78)	97.1 (96.8–97.5)	82.3 (79.8–84.8)	98.4 (98.1–98.6)	77.9 (75.0–80.6)	98.3 (97.9–98.6)	87.2 (84.4–89.9)	23.8 (20.4–27.3)
External validation cohort	Baseline	0.71 (0.68–0.73)	96.8 (96.5–97.2)	73.1 (70.5–75.5)	99.3 (99.2–99.4)	83.3 (81.3–85.5)	97.2 (96.8–97.6)	90.1 (87.7–92.5)	34.7 (31.7–37.7)
	Baseline + TDL	0.72 (0.70–0.75)	96.6 (96.3–97.0)	86.6 (84.8–88.4)	97.9 (97.6–98.1)	71.0 (68.5–73.7)	98.5 (98.1–98.8)	92.0 (89.8–94.2)	44.0 (40.9–47.0)
	Baseline + LRS	0.71 (0.69–0.74)	96.9 (96.6–97.2)	78.2 (75.6–80.4)	98.7 (98.5–98.9)	77.0 (74.2–79.6)	97.9 (97.6–98.2)	92.5 (90.4–94.7)	34.1 (31.0–37.3)
	FFS	0.73 (0.71–0.75)	97.1 (96.7–97.4)	79.0 (76.4–81.1)	98.7 (98.5–98.9)	79.6 (76.9–82.0)	98.1 (97.8–98.4)	94.3 (92.4–96.2)	30.8 (27.9–33.7)

The data in parentheses are 95% confidence interval

*TDL* Temporal difference learning, *LRS* Liver region segmentation, *FFS* Final fusion segmentation, *PPV* Positive predictive value, *NPV* Negative predictive value, *FPR* False-positives ratio

In total, 31 lesions in 21 patients were missed in the internal validation cohort, whereas 13 lesions in 13 patients were missed in the external validation cohort. All missed HCC lesions were small (diameter < 5 cm) and 72.7% of lesions were < 3 cm, which resulted in Dice coefficients for lesions with a diameter ≥ 5 cm and < 5 cm of 0.87 and 0.64, respectively, in the internal validation cohort, and 0.84 and 0.65, respectively, in the external validation cohort. Moreover, 27 patients with multiple lesions resulted in a Dice coefficient of 0.68 in the internal validation cohort, and 30 patients with multiple lesions resulted in a Dice coefficient of 0.67 in the external validation cohort, of whom 64.9% had missed lesions. Among the lesions that had a Dice Coefficient < 0.5, 28.1% had obvious surrounding dynamic stomach and intestine images, 24.3% had obvious motion artifacts of the diaphragm and heart, and 23.2% were small (< 2 cm). Nine patients with an amplified FOV of DSA images achieved a Dice coefficient of 0.79 in the internal validation cohort, and 31 patients with an amplified FOV of DSA images achieved a Dice coefficient of 0.62 in the external validation cohort.

### Performance of treatment response prediction network (model 2)

We further analyzed the segmented lesions and built Model 2 to predict the treatment response to the first TACE session. Model 2 integrated the DSA videos, segmentation results, and clinical variables, achieving an AUC, accuracy, sensitivity, specificity, PPV, and NPV of 78.2% (95% CI: 73.8–82.6), 78.2% (95% CI: 74.2–82.3), 77.6% (95% CI: 70.7–84.0), 78.7% (95% CI: 72.9–84.1), 74.4% (95% CI: 67.2–81.4), and 81.5% (95% CI: 75.9–86.8), respectively, in the internal validation cohort. The generalizability of Model 2 was tested in an independent external validation cohort and reached an AUC, accuracy, sensitivity, specificity, PPV, and NPV of 67.0% (95% CI: 61.2–72.6), 75.1% (95% CI: 70.2–79.5), 50.5% (95% CI: 40.0–61.5), 83.5% (95% CI: 79.2–87.7), 51.1% (95% CI: 40.6–61.4), and 83.2% (95% CI: 78.9–87.5), respectively. Furthermore, a comparison between the different inputs of the predictive model showed that Model 2 had a significantly higher performance than that of original key frames and clinical variables alone (*p* < 0.001). The performance of the predictive models is presented in Table 3.

**Table 3:** Performance of predictive models in the validation cohorts

Cohort	Input	Model	AUC	Accuracy (%)	Sensitivity (%)	Specificity (%)	PPV (%)	NPV (%)
Internal validation cohort	KF	Resnet	0.681 (0.637–0.725)	70.4 (65.3–75.0)	47.3 (39.5–55.2)	88.9 (84.5–93.0)	77.2 (69.1–85.4)	67.9 (61.5–73.5)
	Clinical data	MLP	0.670 (0.623–0.719)	67.7 (62.9–72.6)	60.0 (52.5–67.3)	73.9 (67.6–79.7)	64.7 (56.7–71.8)	69.9 (63.9–75.7)
	KF+KF*Pred+Pred	Resnet	0.733 (0.687–0.779)	73.7 (69.4–78.8)	69.7 (62.8–76.8)	76.8 (70.5–82.2)	70.6 (63.5–77.4)	76.1 (70.1–81.9)
	KF+KF*Pred +Pred+clinical data	Resnet+MLP	0.782 (0.738–0.826)	78.2 (74.2–82.3)	77.6 (70.7–84.0)	78.7 (72.9–84.1)	74.4 (67.2–81.4)	81.5 (75.9–86.8)
	KF+ KF* GT+GT	Resnet	0.727 (0.684–0.770)	74.7 (70.2–78.8)	55.2 (47.4–63.2)	90.3 (86.3–94.1)	82.0 (75.0–89.0)	71.6 (65.9–77.2)
	KF+KF*GT +GT+clinical data	Resnet+MLP	0.802 (0.759–0.847)	80.4 (76.3–84.4)	78.8 (72.2–84.9)	81.6 (76.5–86.6)	77.4 (70.6–83.7)	82.8 (77.4–88.0)
External validation cohort	KF	Resnet	0.628 (0.573–0.684)	69.4 (64.5–74.6)	49.5 (38.6–59.4)	76.2 (71.2–81.5)	41.4 (32.1–50.4)	81.6 (77.0–86.0)
	Clinical data	MLP	0.593 (0.529–0.646)	63.1 (58.5–68.3)	51.6 (42.1–63.1)	67.0 (61.6–72.7)	34.8 (26.8– 42.8)	80.3 (75.0–86.1)
	KF+KF*Pred+Pred	Resnet	0.712 (0.672–0.753)	73.9 (69.6–78.5)	46.7 (39.2–54.2)	95.7 (92.8–98.1)	89.5 (83.0–96.0)	69.2 (63.8–74.5)
	KF+KF*Pred +Pred+clinical data	Resnet+MLP	0.670 (0.612–0.726)	75.1 (70.2–79.5)	50.5 (40.0–61.5)	83.5 (79.2–87.7)	51.1 (40.6–61.4)	83.2 (78.9–87.5)
	KF+KF*GT+GT	Resnet	0.575 (0.536–0.614)	76.2 (71.6–80.6)	19.4 (12.1–28.3)	95.6 (93.0–97.9)	60.0 (41.2–78.3)	77.7 (73.3–81.9)
	KF+KF*GT +GT+clinical data	Resnet+MLP	0.817 (0.777–0.856)	77.9 (73.8–82.0)	89.2 (82.2–95.2)	74.0 (68.1–79.2)	53.9 (46.2–61.8)	95.3 (92.4–97.7)

The data in parentheses are 95% confidence interval

*AUC* Area under the curve, *PPV* Positive predictive value, *NPV* Negative predictive value, *KF* Key frame, Pred: segmentation result from Model 1; GT: segmentation result from ground truth; MLP Multi–layer perceptron

**Fig. 4:**
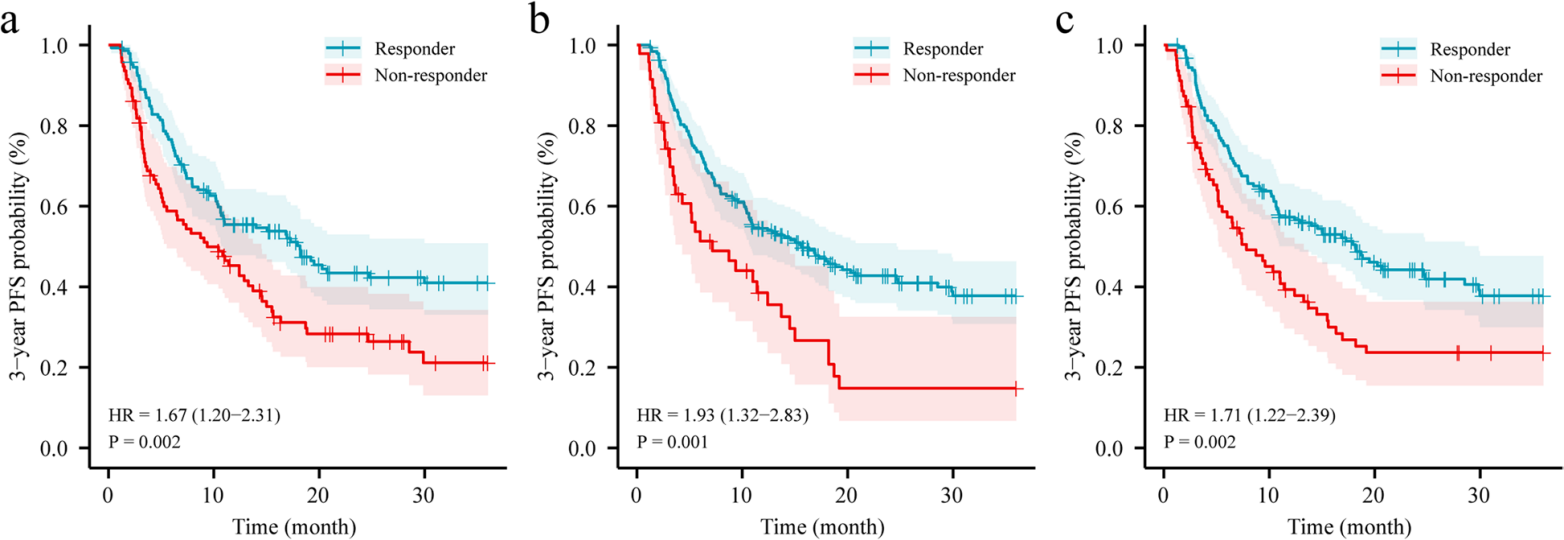
Kaplan-Meier curves of 3-year PFS between the responders and non-responders in the validation cohort. The two response groups were divided by the models constructed by (**a**) clinical data only; (**b**) key frame of DSA videos and segmentation results; and (c) key frame of DSA videos, segmentation results, and clinical data. PFS, progression-free survival; DSA, digital subtraction angiography

As a comparison, the ground truth rather than the segmentation results was input into the model to integrate with the DSA videos and clinical variables, and this yielded an AUC, accuracy, sensitivity, specificity, PPV, and NPV of 80.2% (95% CI: 75.9–84.7), 80.4% (95% CI: 76.3–84.4), 78.8% (95% CI: 72.2–84.9), 81.6% (95% CI: 76.5–86.6), 77.4% (95% CI: 70.6–83.7), and 82.8% (95% CI: 77.4–88.0), respectively, in the internal validation cohort. When tested in the external validation cohort, the model achieved an AUC, accuracy, sensitivity, specificity, PPV, and NPV of 81.6% (95% CI: 77.7–85.6), 77.9% (95% CI: 73.8–82.0), 89.2% (95% CI: 82.2–95.2), 74.0% (95% CI: 68.1–79.2), 53.9% (95% CI: 46.2–61.8), and 95.3% (95% CI: 92.4–97.7), respectively. The performance of the input segmentation results with DSA videos was slightly lower than the input ground truth with DSA videos (*p* > 0.05). Kaplan-Meier analysis (Fig. 4) showed that the 3-year PFS of non-responders was significantly lower than that of the responders (*p* < 0.05).

## Discussion

In this study, we established and validated a clinically-assisted DSA-Net, which included two sub-networks: an automatic segmentation network (Model 1) and a treatment response prediction network (Model 2). Model 1 automatically located HCC lesions on DSA videos. Model 2 integrated the DSA videos, segmentation results, and clinical variables to predict treatment response to the first TACE session and yielded high predictive performance.

In the clinic, DSA prior to TACE directly locates lesions, guides catheters, and evaluates treatment, which further assists clinicians to make future treatment decisions. However, these processes are operator-dependent, which results in interobserver bias between senior and junior clinicians. With the significant advances in medical artificial intelligence, computer analysis of DSA videos allows clinicians to eliminate the potential obstacle of interobserver bias and enables clinicians to deliver precise individualized therapy. To this end, we first detected and segmented HCCs in DSA videos by using classical baseline networks. However, the performance of the baseline models was unsatisfactory. Thus, we explored potential reasons for the complexity of the automatic segmentation tasks. First, the image quality of DSA videos varied, which was related to scanner properties and acquisition parameters. Second, BCLC stage B included multinodular tumors. Under these circumstances, some small lesions were easily missed by traditional CNN networks. Third, although most HCC lesions were hypervascular with obvious staining in DSA videos, some lesions were hypovascular with light and blurred staining that were difficult to detect. Finally, the movement interference surrounding the image of the liver, which mainly included the image of the stomach and intestine, and motion artifacts of the diaphragm and heart may have caused false segmentation. For these reasons, the detection and classification of DSA videos using traditional DL methods have challenges. To date, to the best of our knowledge, there have not been any reports of using the DL approach for decoding DSA videos of tumors.

Here, we referred to the method used by clinicians to detect HCCs in DSA videos, which considers HCC a dynamic staining process in DSA videos, and all tumors are in or linked to liver regions. We designed two specific steps to improve the accuracy of the segmentation model. The first step was TDL based on 10 frames, and the second was to learn liver region segmentation to narrow the area of detection. Integration of these two steps significantly enhanced the accuracy of the segmentation model and achieved real-time segmentation with a video processing time of 10 ms. Additionally, the generalization of automatic segmentation was validated in an independent cohort and offered an opportunity to rapidly locate HCCs and avoid missing multinodular tumors during TACE therapy. Moreover, the subgroup analysis of Model 1 showed that the segmentation model was highly reliable for larger lesions (≥ 5 cm) or images with fewer than two lesions, whereas motion artifacts, surrounding interference images, and multiple small lesions (< 3 cm) were key factors that contributed to false segmentation. Furthermore, because of the different procedures used by operators, different FOVs of DSA videos increased segmentation difficulties. Thus, the standard operation of the technical procedures contributes to the real-world application of Model 1.

When lesions are segmented, the heterogeneity of the tumor areas is further decoded to identify underlying information to assist in individualized clinical management. The success of TACE relies on determining whether a patient will benefit from TACE. Model 2 could classify patients into responder or non-responder groups before chemoembolization. Patients classified as responders to the first TACE session may be ideal candidates for TACE therapy. Notably, several previous studies have shown that patients who showed no response to the first TACE session and received further TACE therapy achieved an objective response and similar survival outcomes to those who responded to the first TACE session [25, 26]. However, a global non-interventional prospective study called OPTIMIS showed that after repeated TACE therapy, the objective response rate gradually decreased, whereas the progressive disease rate increased [27]. Recent evidence has also indicated that when patients progress, their liver function significantly decreases; moreover, switching to sorafenib is difficult [28]. Our survival analysis suggested that compared with responders, non-responders had a poorer prognosis and may not benefit from further TACE sessions. Thus, other evidence-based treatments, such as ablation or systemic therapy combined with TACE, should be strongly recommended for non-responder groups.

This study has several limitations. First, this was a retrospective study; thus, some bias between the medical record system and real practice is inevitable. Thus, further prospective multicenter studies are needed to optimize the performance of DSA-Net. Second, although CT- or US-based models have better performance for predicting treatment response than do DSA-based models, the DSA-based model can directly and rapidly determine arterial blood supply, which may allow clinicians to adjust therapeutic schedules promptly. Furthermore, we found that better segmentation results improved the performance of the predictive models, and the performance of Model 2 can be improved further than that of the model based on the ground truth. Thus, the DSA-based model still offers higher prediction performance than the current accuracy of segmentation by further integrating pathological results with the tumor microenvironment. Third, the majority of Chinese HCC patients have chronic HBV infection, whereas 86% of the HCC patients enrolled in this study had HBV infection. Our results showed that HBV infection was associated with treatment response, which was consistent with previous studies that demonstrated that HBV infection affects HCC therapy treatment response and prognosis [29]. Additionally, the major risk factors for HCC vary across different regions, such as alcohol abuse, obesity, and type-2 diabetes, which are considered predominant causes of HCC in other countries [30]. Thus, large-scale external validations in HBV endemic and non-endemic areas are necessary. Finally, to eliminate the influence of confounding factors, several patients with BCLC stage B who received chemoembolization with drug-eluting beads were not included. Hence, to determine the real-world clinical benefit of DSA-Net for automatic tumor segmentation and prognostic prediction, we plan to conduct a multi-therapy trial.

## Conclusions

DSA-Net enabled automatic detection and segmentation of HCCs during TACE, which may aid clinicians to locate lesions rapidly. DSA-Net may provide clinical-decision support by dividing HCC patients into two treatment response groups with diverse prognosis. Thus, DSA-Net may be a useful predictive tool for identifying patients who would benefit from TACE and providing a basis for clinical recommendations of TACE. For clinicians to fully accept and confidently apply the model to patient management, further validation studies in patients with different etiologies from different endemic areas are highly warranted.

## Supplementary Information

**Additional file 1.**

## Data Availability

The data are not available for public access because of patient privacy concerns, but are available from the corresponding author on reasonable request.

## Abbreviations

HCC: Hepatocellular carcinoma
TACE: Transcatheter arterial chemoembolization
DSA: Digital subtraction angiography
DL: Deep learning
mRECIST: Modified Response Evaluation Criteria in Solid Tumors
CR: Complete response
PR: Partial response
SD: Stable disease
PD: Progressive disease
TDL: Temporal difference learning
LRS: Liver region segmentation
FFS: Final fusion segmentation
