# Supplementary material for: Real-time automatic prediction of treatment response to transcatheter arterial chemoembolization in patients with hepatocellular carcinoma using deep learning based on digital subtraction angiography videos
Source: Cancer Imaging. 2022 May 12;22:23. doi: 10.1186/s40644-022-00457-3 (PMC9101835; doi:10.1186/s40644-022-00457-3)
Supplement: Supplementary file 1 — Additional file 1. [file 40644_2022_457_MOESM1_ESM.docx]

**Supplementary Materials**

**Supplementary Methods**

***S1: The acquisition parameters of DSA***

Digital subtraction angiography (DSA) videos were obtained under the situation that patients held their breath, whose frame rate was 2-4 frames/s. Contrast medium was injected at 2-6 ml/s with a Mark V ProVis injector (Medrad, Warrendale, PA, USA). 5F or 4F introducer sheath (Terumo Europe, Interleuvenlaan, Belgium) and 2.7F microcatheter (Renegade^TM^ HI-FLO^TM^ with Transend^TM^ 18 guidewire, Boston Scientific, Marlborough, MA, USA, or Merit MaestroTM with TenorTM 0.014 guidewire, Merit Medical Systems, Utah, USA) were used. The medical equipment used in 2 different hospitals for DSA video acquisition were from either Siemens Healthcare (Germany) or Philips Healthcare (Netherlands). These equipments were all set to 50mAs/120kV for anteroposterior projections when the radiologists from both hospitals shot the DSA videos. The visible resolutions of DSA videos varied according to clinical needs, which included 1024×1024, 1920×1080, and1092×2480.

***S2: Development of DSA-Net***

We chose cross-entropy loss and Dice loss as the segmentation loss. Each model was trained for 150 epochs from scratch using a NVIDIA GTX 8119MiB GPU. The models were implemented with PyTorch. We used Adam optimizers to update the network parameters. The initial learning rate was set to 0.001 and the batch size was set to 8. Because the location of tumors in DSA videos are relatively fixed, traditional data augmentation methods such as rotation, scaling and flipping were not applied on DSA images.

For automatic selection of key frames, we designed the following method. We ﬁrstly calculated the diﬀerence images between 2 adjacent frames in the last 15 frames of DSA videos. To judge whether a frame was the key frame, we averaged the pixel values of the 2 diﬀerence images before and after the frame. We selected the frame with the minimum average pixel value as the key frame because the imaging in the key frame was the most stable one in the frames, which appeared as the minimum average pixel value.

***Development of Model 1***

The proposed temporal diﬀerence learning (TDL) with multi-frame inputs was adopted for auto-learning temporal diﬀerence under the supervision of interframe diﬀerence images. The TDL module concatenated the consecutive frames (key frame, *k*-1, *k*-2…*k*-9) as its input and used an independent U-Net to learn the latent motion information from the input. Using the auto-learned results under the supervision of frame diﬀerences (FD) instead of using the frame differences directly can help the module dig out more useful motion information. The inter-frame differences were calculated between the (*k*-9)-th and the key frame (*k*-th) for the purpose of acquiring the biggest difference from the key frame. Because of the limitation of video frame amount, we chose the maximum of 10 frames. It was worth recalling that, using the pixel-based difference between the two consecutive frames could reflect the objects that gradually appeared more apparently than other methods, such as optical flow and background subtraction difference. The absolute differential image is defined as follows:

*I_d (k, k+1)_ = |I_k+1_-I_k_|*

where *I_k_* and *I_k+1_* are the values of the *k*-th and (*k* + 1)-th frames in image sequences, respectively. As shown in **Figure 2**, the FD captured the changing information of tumor area across frames efficiently, especially when it came to the furthest temporal distance.

Our method also integrated prior anatomical knowledge of liver regions as guidance. We designed a LRS sub-network based on U-Net, which is co-trained on the key frames to predict the liver regions. The predicted liver region masks (LM) produced by LRS guided our model in locating tumors, accurately.

***Development of Model 2***

The ResNet module was pre-trained on ImageNet and fine-tuned on our dataset for 200 epochs with Adam optimizers. The Multi-layer perceptron (MLP) branch was trained from scratch with the tubular data. The batch size was set to 32 and the initial learning rate was set to 0.01. From those 200 epochs, the best model was selected via accuracy performance on the validation set.

For the image branch, in addition to the key frames (256 × 256 × 1) and segmented results (256 × 256 × 1) from Model 1, the masked key frames from the segmented results were designed to help the CNN subnet pay more attention to the tumor areas. The three images were concatenated and input into the CNN subnet for image feature extraction. For the tabular data branch, all clinical data were processed before inserted into the MLP, which could help extract features more easily. The discrete data were encoded into one-hot format. The continuous values were also changed into one-hot format according to the cutoffs given by the clinical value or mean. Moreover, the relative positions where the values placed in the duration were added. The relative positions were calculated as follows: position = (value – mid)/duration length. For example, if a value 10 and two cutoffs [4,12] are given, the cutoffs will construct 3 durations and the value will fall in the second duration. Therefore, its position will be equal to (10 – 8)/ (12-4) =0.25 and the continuous data, 10, will become [0,1,0,0,25]. The Model 2 was constructed by integrating tumor patches and clinical parameters to predict treatment response. The output of Model 2 was also in one-hot format [P(label=0), P(label=1)], and the Argmax function picked up the bigger probability to generate the final label, which divided patients into: responder and non-responder groups (0,1). The selected model was then evaluated on the internal and external validation cohort, where the reported accuracy performance values were obtained.

**Supplementary tables**

**Table S1. Performance of baseline models in the internal validation cohort**

| Baseline model | Dice | Accuracy | Sensitivity | Specificity | PPV | NPV |
| --- | --- | --- | --- | --- | --- | --- |
| U-net | 0.71  (0.68–0.74) | 96.8  (96.4–97.2) | 80.2  (77.6–82.7) | 98.2  (97.8–98.5) | 75.5  (72.3–78.5) | 98.1  (97.8–98.4) |
| U-net ++ | 0.71  (0.68–0.74) | 96.8  (96.5–97.2) | 80.6  (78.0–83.0) | 98.2  (97.9–98.5) | 74.0  (70.8–77.1) | 98.1  (97.8–98.4) |
| Attention U-Net | 0.71  (0.69–0.74) | 96.7  (96.3–97.1) | 80.8  (78.0–83.5) | 98.0  (97.7–98.3) | 74.6  (71.5–77.8) | 98.2  (97.9–98.5) |
| nnU-net | 0.72  (0.70–0.74) | 98.3  (98.0–98.5) | 86.6  (84.2–89.0) | 99.1  (98.9–99.3) | 86.9  (84.5–89.2) | 98.9  (98.6–99.1) |
| U^2^-Net | 0.69  (0.67–0.72) | 96.0  (95.6–96.5) | 85.7  (83.1–88.3) | 96.5  (95.9–96.9) | 66.5  (63.0–69.6) | 98.9  (98.8–99.1) |

Note: The data in parentheses are 95% confidence interval. PPV, positive predictive value; NPV, negative predictive value.

**Table S2. Performance of segmentation model with different frames in the internal validation cohort**

| Number of frames | model | Dice | Accuracy | Sensitivity | Specificity | PPV | NPV |
| --- | --- | --- | --- | --- | --- | --- | --- |
| 6 | Baseline + TDL | 0.71  (0.68–0.74) | 96.6  (96.2– 97.0) | 82.6  (79.8–85.0) | 97.5  (97.1–97.9) | 70.6  (67.3–73.6) | 98.5  (98.2–98.8) |
| 8 | Baseline + TDL | 0.71  (0.69–0.74) | 96.7  (96.4–97.1) | 79.7  (77.2–82.2) | 98.2  (97.9–98.5) | 75.2  (71.9–78.2) | 98.0  (97.7–98.3) |
| 10 | Baseline + TDL | 0.72  (0.70– 0.75) | 96.7  (96.3–97.0) | 83.3  (81.0–85.6) | 97.7  (97.3–98.0) | 73.4  (70.2–76.3) | 98.4  (98.2–98.7) |
| 12 | Baseline + TDL | 0.72  (0.70–0.75) | 96.4  (96.0–96.8) | 84.1  (81.9–86.2) | 97.4  (97.0–97.8) | 72.3  (69.2–75.4) | 98.3  (98.1–98.6) |
| 14 | Baseline + TDL | 0.71  (0.68–0.73) | 96.7  (96.3–97.0) | 79.4  (76.9–81.8) | 98.0  (97.7–98.4) | 74.0  (70.8–77.1) | 98.1  (97.8– 98.4) |

Note: The data in parentheses are 95% confidence interval. TDL, temporal diﬀerence learning; PPV, positive predictive value; NPV, negative predictive value.
